# A Critical Analysis of Pharyngeal Patterns of Collapse in Obstructive Sleep Apnea: Beyond the Endoscopic Classification Systems

**DOI:** 10.3390/jcm13010165

**Published:** 2023-12-27

**Authors:** Andrea De Vito, Ewa Olszewska, Bhik Kotecha, Eric Thuler, Manuele Casale, Giovanni Cammaroto, Claudio Vicini, Olivier M. Vanderveken

**Affiliations:** 1 ENT Unit, Department of Surgery, Ravenna-Lugo Hospitals, Health Local Agency of Romagna, 48121 Ravenna, Italy; dr.andrea.devito@gmail.com; 2 ENT Unit, Department of Surgery, Forlì—Faenza Hospitals, Health Local Agency of Romagna, 47122 Forlì, Italy; 3 Department of Otolaryngology, Medical University of Bialystok, 15-328 Bialystok, Poland; ewa.olszewska@umb.edu.pl; 4 Queens Hospital, Barking Harvering and Redbridge University Hospitals NHS Trust, Rom Vally Way, Romford RM1 2BA, UK; bhikkot@aol.com; 5 Sleep Surgery Division, OHNS Department, University of Pennsylvania, Philadelphia, PA 19104, USA; erthuler@gmail.com; 6 Integrated Therapies in Otolaryngology, Fondazione Policlinico Universitario Campus Bio-Medico, 00128 Rome, Italy; m.casale@policlinicocampus.it; 7 ENT Unit, Faenza Hospital, Villa Maria Group, 48018 Faenza, Italy; claudio@claudiovicini.com; 8 Department of Otorhinolaryngology-Head and Neck Surgery, Antwerp University Hospital (UZA), 2650 Antwerp, Belgium; olivier.vanderveken@uza.be; 9 Faculty of Medicine and Health Sciences, University of Antwerp, 2610 Antwerp, Belgium

**Keywords:** obstructive sleep apnea, drug-induced sleep endoscopy, DISE classification scoring systems, VOTE classification, upper airways surgery

## Abstract

(1) Background: Drug-Induced Sleep Endoscopy (DISE) enables the three-dimensional and dynamic visualization of the upper airway (UA) during sleep, which is useful in selecting the best treatment option for obstructive sleep apnea (OSA) patients, particularly for surgical procedures. Despite international consensus statements or position papers, a universally accepted DISE methodology and classification system remain a controversial open question. (2) Methods: A review of the English scientific literature on DISE related to endoscopic classification systems and surgical outcome predictors (3) Results: Of the 105 articles, 47 were included in the analysis based on their content’s relevance to the searched keywords. (4) Conclusions: A final report and scoring classification system is not universally accepted; the most internationally applied endoscopic classification system during DISE does not cover all patterns of events that occur simultaneously during the endoscopic examination, highlighting that several configurations of collapse and obstruction at different UA levels could be observed during DISE, which should be described in detail if DISE has to be considered in the decision-making process for the UA surgical treatment in OSA patients and if DISE has to have a role as a predictive factor for surgical outcomes analysis.

## 1. Introduction

Obstructive sleep apnea (OSA) is the most common and under-diagnosed sleep-disordered breathing (SDB) disease, with a high prevalence in the adult population, which is reported from 13% to 33% in men and 6% to 19% in women, considering the methodological heterogeneity of the epidemiological studies [1]. The pathophysiology of OSA is quite complex and is related to specific pathophysiological traits: high upper airway (UA) collapsibility, UA muscular impairment, unstable ventilatory control system or loop gain (LG), and low arousal threshold (AT). Even though the pharyngeal collapsibility (anatomical factor) is the most important factor amongst the PTs, in more than 70% of OSA patients, it could be a relative contribution or combination of each of the anatomical and non-anatomical PTs [2].

Pharyngeal collapsibility is measured by the passive critical closing pressure (Pcrit), where a high Pcrit (>2 cm H_2_O) is related to high pharyngeal collapsibility [2]. Continuous Positive Airway Pressure (CPAP) represents the first-line treatment option for OSA, but with a long-term decrease in compliance from 50 to 70%. Consequently, alternative treatment options are being advocated, such as UA surgery, mandibular advancement devices (MAD), and positional therapy [2,3].

The main result achievable with surgical treatment is the remodeling of UA, with the expansion and/or stabilization and/or tissue removal to different UA levels, which determine the improvement of UA resistance to collapse during obstructive sleep events. In this view, a targeted approach based on medical history, sleep studies, and clinical examination is of pivotal importance for the detection of the presence and preponderance of anatomical factors in UA collapsibility and for the selection of the best surgical procedures for each OSA patient [4].

Drug-induced Sleep Endoscopy (DISE) represents one of the most widespread diagnostic procedures for the identification of UA anatomical sites of obstruction in OSA patients, classifying the findings according to the severity and configuration of UA collapse [1]. DISE methodology, feasibility, and reliability have been extensively investigated since its first introduction by Pringle and Croft in 1991, as demonstrated by the widely published literature data [1,5,6]. There is general agreement that DISE enables analysis of the non-REM N2 sleep stage and can provide important dynamic and three-dimensional views of the main sites of UA collapse during apnea events [7].

A comparison between DISE and natural sleep endoscopy (NSE) reported an overall agreement between DISE and NSE findings [8]. Consequently, DISE may be of pivotal importance in the selection and customization of candidates for UA surgical treatment, as well as MAD, hypoglossal nerve stimulation, and the analysis of Continuous Positive Airway Pressure (CPAP) failure [6,9,10].

Despite the VOTE (Velum-Oropharyngeal-Tongue-Epiglottis) classification seeming to be the most adopted scoring system, the lack of a universally accepted interpretation and classification of several DISE findings remains the main critical open question and may explain inconsistencies in the predictive value for surgical outcomes [11].

The aim of this paper is to review the current literature for DISE, analyze the findings presented by the most widely adopted classification system, and highlight the need to assess not only the site and degree of the obstruction but also the type and pattern of collapse as well as the palatal phenotype during DISE to play a role as a predictive factor for surgical outcomes in OSA patients.

## 2. Methods

Comprehensive bibliographic research was performed on PubMed to report the most updated and comprehensive standardized literature data on DISE applied to the OSA adult patients’ population. The literature search was conducted and focused mainly on meta-analyses, systematic reviews, and randomized controlled trials (RCT) published in the English language from 2012 to June 2023. The main keywords, or its acronym (drug-induced sleep endoscopy, DISE), were combined with the use of the “AND” function to better select the research with the following keywords: 1/clinical application, 2/endoscopic technique, 3/pharyngeal patterns of collapse, 4/scoring classification system, and 5/surgical outcomes. When systematic reviews, meta-analyses, and RCTs were not available, updated international statements or position papers were obtained. Single original articles were also cited if they were encountered in the analysis of systematic reviews, meta-analyses, and RCTs and were considered significant for the present study.

## 3. Results

A total of 105 papers were found using the reported keywords. Approximately 54 studies were identified that included only systematic reviews, meta-analyses, and RCTs as keywords. Therefore, excluding overall reviews resulted in approximately a 50% decrease in scientific literature. Furthermore, one international statement and two position papers were identified. A final selection of 46 papers was generated based on their content’s relevance to the searched keywords (Table 1).

### 3.1. DISE and UA Pattern of Collapse/Obstruction (Table 2)

During DISE, it is possible to visualize different patterns and grades of UA collapse and obstruction at different levels and areas of the UA: at the soft palate, at the oropharyngeal lateral walls, at the base of the tongue, at the epiglottis, at the oral tongue, and at the nasal cavities. The patterns might be summarized as follows:

**Table 2:** DISE descriptive classification.

UA Site	UA Pattern of Collapse	
Patterns of Soft Palate Collapse	concentric collapse	- primary - simultaneous latero-lateral and antero-posterior collapse
	antero-posterior collapse	- primary - secondary to antero-posterior collapse of a vertically positioned muscular tongue base
Patterns of Oropharyngeal Lateral Walls	latero-lateral collapse	- oropharyngeal primary latero-lateral collapse - oropharyngeal obstruction due to palatine tonsil hypertrophy - mixture pattern
Patterns of Base of Tongue Collapse	antero-posterior collapse	- obstruction for lingual tonsil hypertrophy - antero-posterior collapse of a vertically positioned muscular tongue base
Patterns of Epiglottic Collapse	antero-posterior collapse latero-lateral collapse	- primary, “trapdoor” phenomenon - secondary due to obstruction of lingual tonsil hypertrophy - secondary due to antero-posterior collapse of a vertically positioned muscular tongue base - secondary due to the aryepiglottic fold pattern of collapse

#### 3.1.1. Patterns of Soft Palate Collapse

##### Concentric Pattern

**Figure 1:**
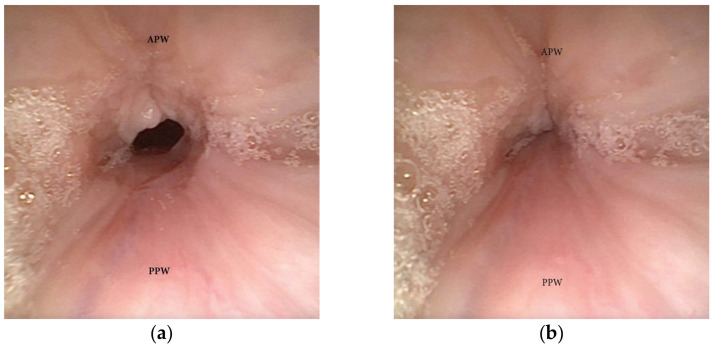
(**a**,**b**) DISE pattern of complete concentric collapse (CCC) of the soft palate. (APW: anterior pharyngeal wall; PPW: posterior pharyngeal wall).

**Figure 2:**
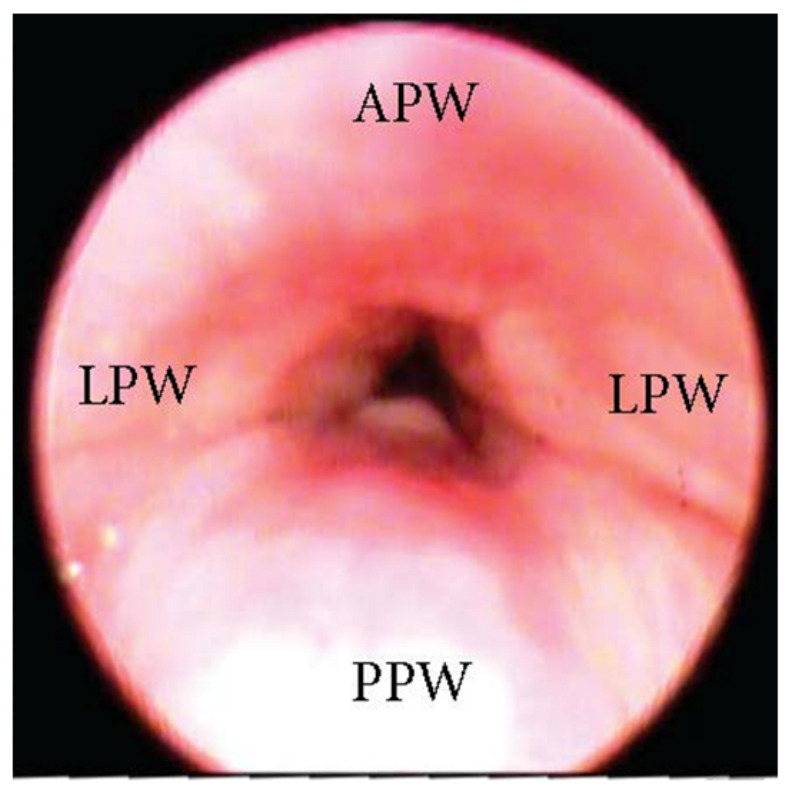
DISE pattern of latero-lateral/antero-posterior collapse of the soft palate. (APW: anterior pharyngeal wall; PPW: posterior pharyngeal wall; LPW: lateral pharyngeal wall).

One of the most clinically significant patterns of collapse visualized at the soft palate during DISE is defined as concentric (Figure 1), and it is referred to as the primary complete concentric collapse (CCC) DISE pattern. Otherwise, Van de Perck et al. [12] reported that in some OSA patients, careful observation of the CCC pattern during DISE could detect a simultaneous latero-lateral and antero-posterior collapse (LL-AP) at the velum level (Figure 2).

##### Antero-Posterior (AP) Collapse

**Figure 3:**
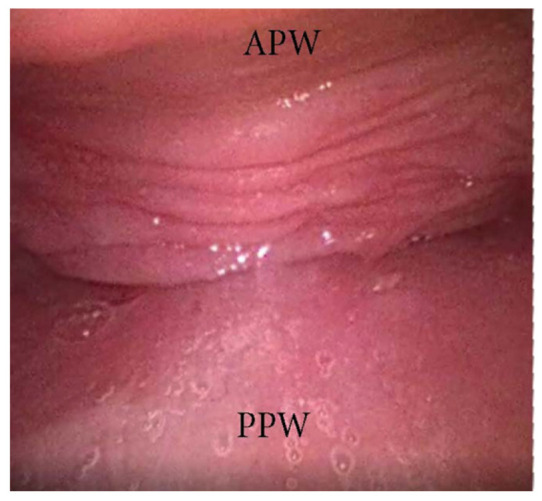
DISE pattern of primary antero-posterior collapse of soft palate. (APW: anterior pharyngeal wall; PPW: posterior pharyngeal wall).

**Figure 4:**
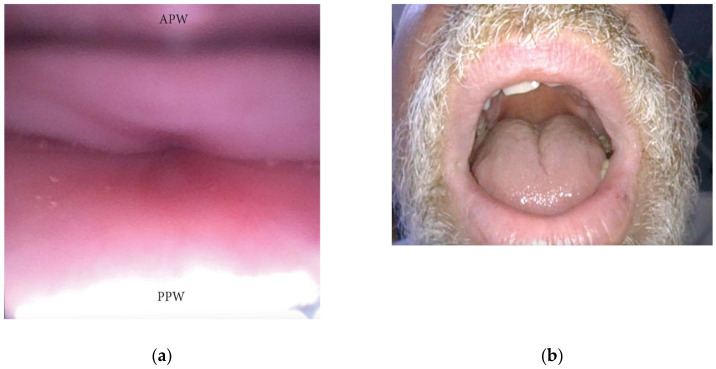
(**a**,**b**) DISE pattern of secondary antero-posterior collapse of the soft palate due to the posterior displacement of the junctional tongue (APW: anterior pharyngeal wall; PPW: posterior pharyngeal wall).

An AP collapse at the soft palate level during DISE can be defined as primary if it is due to an intrinsic collapse modality of the soft palate (Figure 3). However, in a significant percentage of OSA patients, a soft palate AP collapse can be driven by the posterior displacement of the junctional tongue (Figure 4). Furthermore, in the case of primary AP palatal collapse, the analysis of the soft/hard palate angle is also of pivotal importance, as a primary AP pattern of collapse is usually associated with an acute angle in which the posterior pharyngeal wall is a short distance from the soft palate [13].

#### 3.1.2. Patterns of Oropharyngeal Lateral Walls

**Figure 5:**
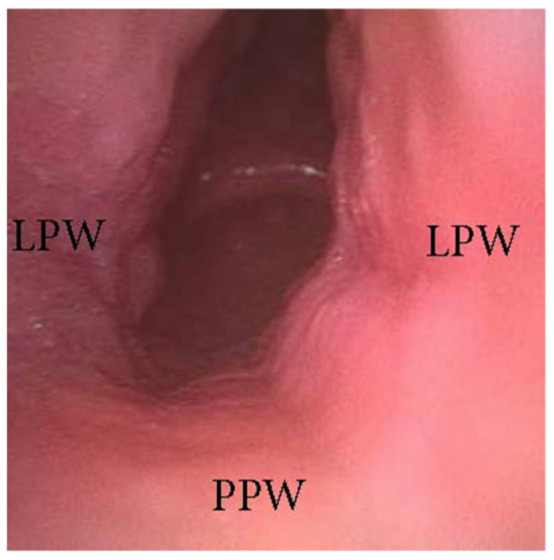
DISE pattern of latero-lateral collapse of the lateral oropharyngeal wall (PPW: posterior pharyngeal wall; LPW: lateral pharyngeal walls).

**Figure 6:**
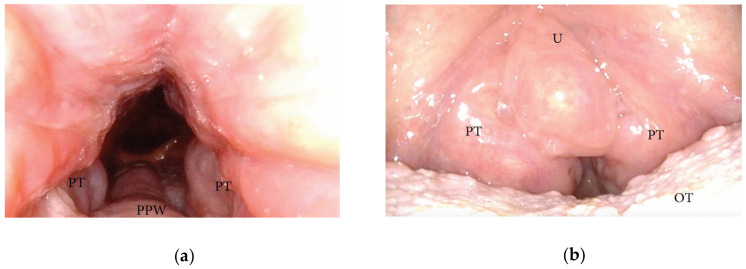
(**a**,**b**) DISE pattern of lateral oropharyngeal wall obstruction due to tonsillar hypertrophy (PPW: posterior pharyngeal wall; PT: palatine tonsils; U: uvula; OT: oral tongue).

The oropharyngeal wall collapse frequently assumes a latero-lateral pattern during DISE, which is often associated with a more severe pharyngeal collapsibility (Figure 5). Otherwise, in adult OSA patients, the presence of palatine tonsil hypertrophy also results in partial or complete latero-lateral occlusion of the oropharynx during apnea events (Figure 6). Furthermore, in most OSA patients, a latero-lateral pharyngeal collapse may merge both palatine tonsil hypertrophy and a more severe extrinsic oropharyngeal lateral wall collapse.

#### 3.1.3. Patterns of the Base of Tongue Collapse

**Figure 7:**
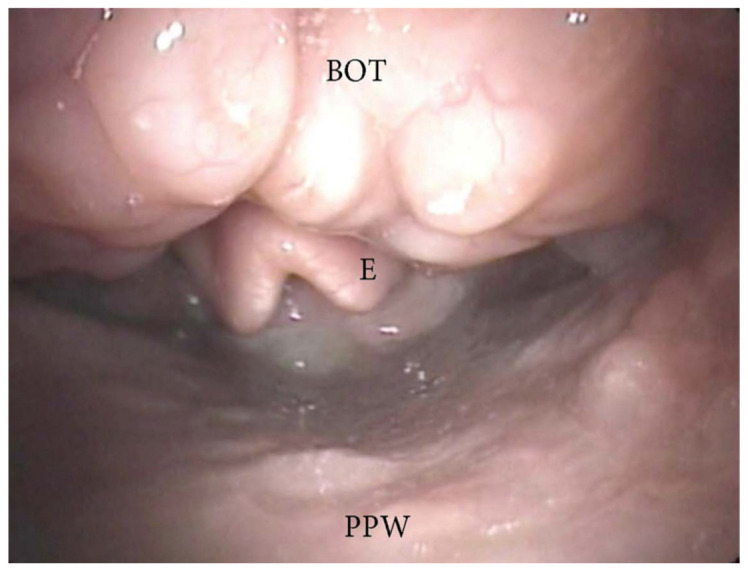
DISE pattern of obstruction due to lingual tonsil hypertrophy (BOT: base of the tongue; PPW: posterior pharyngeal wall; E: epiglottis).

**Figure 8:**
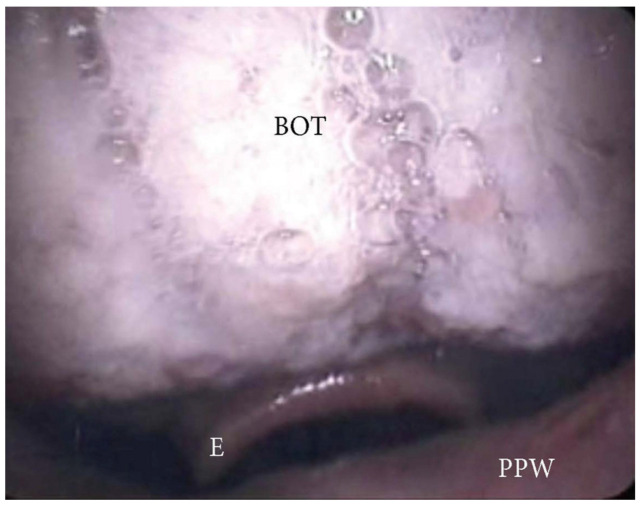
DISE pattern of vertically positioned muscular base of the tongue (BOT: base of the tongue; PPW: posterior pharyngeal wall; E: epiglottis).

The collapse at the level of the base of the tongue can be related to a higher degree of lingual tonsil hypertrophy (Figure 7) or an antero-posterior collapse of a vertically positioned muscular tongue base (Figure 8).

#### 3.1.4. Patterns of Epiglottic Collapse

**Figure 9:**
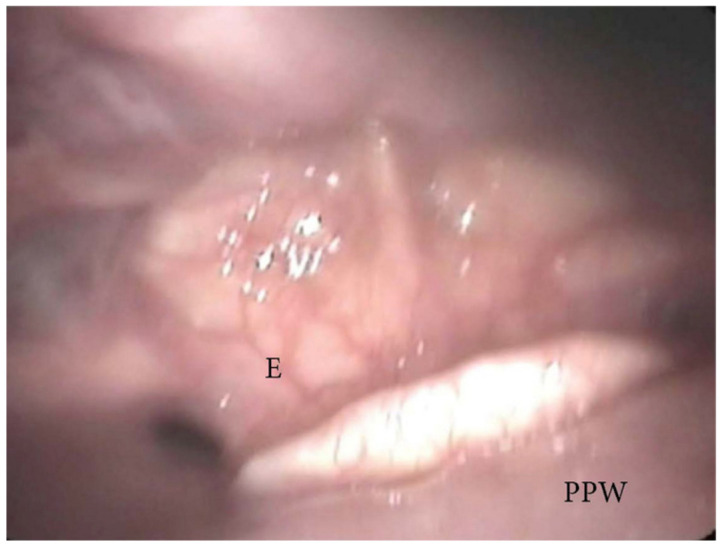
DISE pattern of epiglottic trapdoor collapse (PPW: posterior pharyngeal wall; E: epiglottis).

**Figure 10:**
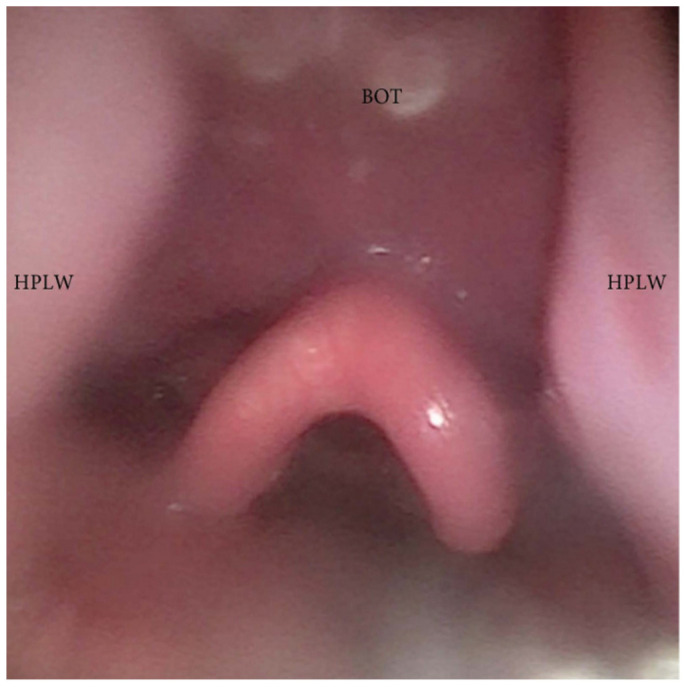
DISE pattern of hypopharyngeal latero-lateral collapse (HPLW: hypopharyngeal lateral wall; BOT: base of the tongue).

**Figure 11:**
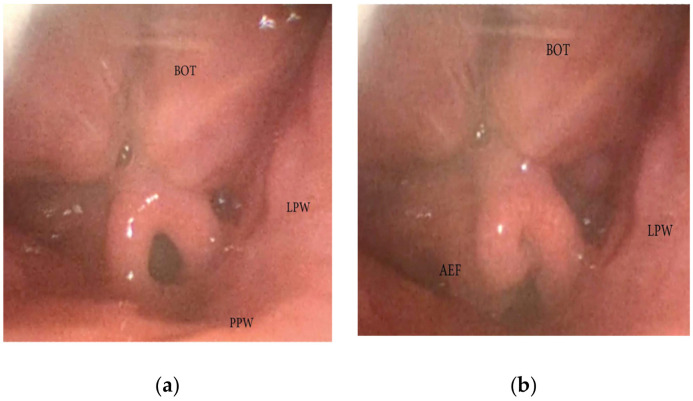
(**a**,**b**) DISE pattern of aryepiglottic collapse (BOT: base of the tongue; PPW: posterior pharyngeal wall: AEF: aryepiglottic fold; LPW: lateral pharyngeal wall).

During DISE, it is possible to analyze a primary anteroposterior configuration of the collapse of the epiglottis, also known as the “trapdoor” phenomenon (Figure 9). More frequently, different secondary collapses are observed due to hypopharyngeal latero-lateral occlusion (Figure 10) or obstruction caused by lymphatic tissue hypertrophy at the base of the tongue (Figure 7). Additionally, an aryepiglottic fold pattern of collapse can be observed, even in the adult OSA population, similar to laryngomalacia in the pediatric population (Figure 11).

#### 3.1.5. Oral Tongue Patterns of Collapse

During DISE, the collapse of the oral tongue should also be analyzed through the mouth. In a significant percentage of OSA patients, a relative disproportion between the oral cavity and the tongue volume can be visualized, leading to posterior displacement of the junctional tongue and a partial or complete obstruction of the soft palate. This finding is suggestive of a tongue-driven anteroposterior soft palate collapse during DISE (Figure 4).

#### 3.1.6. Nasal Obstruction and Pattern of Collapse

The nasal cavity is usually described as the entrance (rigid portion) of the Starling resistor model, which explains the differential pressure between nasal input pressure, pharyngeal surrounding pressure, and laryngotracheal output pressure [14]. While the nose can be considered the rigid segment in this model, there may be a different degree of collapse at the level of the nasal valve area. Additionally, the presence of septal nasal deviation, inferior turbinate hypertrophy, sino-nasal polyposis, or concha bullosa can cause significant nasal obstruction, leading to an increase in a varying degree of negative intraluminal pharyngeal pressure.

## 4. Discussion

The complexity of OSA pathophysiology consists of different endotypes, which need to be identified to achieve an effective multimodal therapeutic approach. Anatomical factors predisposing to UA obstruction during sleep represent the most important, but not the single OSA pathophysiological factor. Eckert et al. found that more than 70% of OSA patients’ non-anatomical factors (high LG, low AT, and neuromuscular impairment) could contribute to the partial or complete UA occlusion [15].

A hyper-responsive ventilation control (high LG) determines a willingness to the ventilatory overshoot response for a minimal CO_2_ perturbation during sleep, resulting in excessive removal of CO_2_ and triggering a situation of cyclical apneic events. A low AT determines respiratory overshoots, which increases ventilator instability control and pharyngeal muscular instability and leads to the recurrence of the obstructive events. Moreover, a low AT could cause sleep fragmentation, contributing to daytime pathologic sleepiness.

Finally, several mechanisms of damage to UA afferent and efferent nerves have been identified and can cause a reduction in the neuromuscular compensation activity of UA muscles during sleep [2,15].

Currently, OSA therapy includes multiple treatment options, which could be conducted at the same time or along the way, and which must be customized to provide the most effective outcomes. Medical history, sleep studies, and awake and sleep clinical examinations represent the armamentarium of the surgeon, which must be applied for the selection of UA surgical therapy candidates with the main purpose of detecting if the anatomical factors are predominant or suitable for a specific surgical procedure.

In this context, DISE represents a complementary exam performed worldwide in sleep centers that offer PAP-alternative treatment (Table 3) [16]. A significant level of reliability and interobserver agreement is reported in the literature for DISE [6,9], allowing a dynamic assessment of the UA obstructive sites occurring during hypopnea and apnea events. Even though many different classification scoring systems were described, the examiner’s experience can significantly improve the interpretation of DISE findings, and there is agreement among authors that DISE determines common patterns of UA collapse, which may be insightful for the underlying causal mechanism [10].

Recently, a European position paper introduced a methodological approach to DISE, achieving the panel’s agreement on DISE indications and contraindications, required preliminary examinations, patient selection, adequate location to perform the exam, essential technical equipment, staffing, patient positioning, sedative drugs available during the endoscopy, the observation window characteristics, the potential useful maneuvers performed, and the main patterns of collapse observed during DISE [7].

However, the DISE scoring and classification system remain controversial. Recently, a meta-analysis reported at least 19 different DISE classification systems with different objective quantitative and semiquantitative features [19,20]. In the European position paper, the working group suggested some general characteristics, such as the level, the degree, and the pattern of the obstruction, which should characterize any DISE classification system, and adopted the VOTE classification for its simplicity and high inter-rater agreement [7]. Likewise, the latest international consensus statement on obstructive sleep apnea [1] adopted VOTE as the official DISE classification system. The VOTE classification reported some specific structures that could contribute to OSA UA obstruction (V: palatine velum, O: oropharyngeal lateral wall, T: base of the tongue, and E: epiglottis), three specific patterns of collapse configuration (A-P: antero-posterior collapse, latero-lateral collapse, and concentric collapse), and two specific degrees of UA obstruction (partial or complete) [11]. However, as specifically reported in the present study, the segmentation of the UA and the subjective interpretation of partial (hypopnea) or complete (Apnea) UA occlusion are considered limitations of VOTE classification, which may compromise the ability of DISE to improve treatment outcomes. Recently, objective measures of UA collapsibility were described during DISE with the addition of PAP titration, upgrading the information gathered by this exam [21].

Therefore, because of those limitations, it has been recommended to leave enough room for additional descriptions of specific DISE findings in the final report (Table 1).

- Velum and Oropharyngeal patterns of collapse:

The distinction between CCC and LL-AP collapse in the velum region is crucial. This is because pure concentric collapse is a contraindication for hypoglossal nerve stimulation [22,23], whereas the LL-AP could take into consideration hypoglossal nerve stimulation as a treatment option. Similarly, differentiating primary vs. secondary tongue-driven antero-posterior palate collapse can significantly impact the outcomes of non-CPAP treatment options. Primary palatal collapse may require surgical therapy addressing the soft tissue or bone dimension (pharyngoplasty techniques), while secondary collapse may benefit from mandibular advancement devices or hypoglossal nerve stimulation. Furthermore, analyzing the soft/hard palate angle is critical in cases of primary AP palatal collapse. An acute angle often correlates with a short distance between the soft palate and posterior pharyngeal wall, making pharyngeal surgical techniques focused on palate-pharyngeal soft tissue remodeling contraindicated due to their low percentage of positive outcomes [13,24]. Furthermore, DISE can include the observation of the salpingopharyngeal fold, suggesting an increased UA collapsibility associated with negative effort dependence and lateral wall collapse, which may change the surgical planning and outcomes for OSA treatment [25].

- Tongue patterns of collapse:

The distinction between a tongue base obstruction due to different grades of lymphatic tissue hypertrophy, with or without secondary epiglottic collapse, and a tongue base collapse of a vertically positioned muscular base of the tongue guides the surgical approach through tongue base reduction (transoral robotic or coblation techniques) in the first case or the use of a mandibular advancement device, skeletal surgery, or hypoglossal nerve stimulation in the second case [7,26,27].

- Epiglottis patterns of collapse:

During DISE, it is possible to observe different configurations of epiglottis collapse, including the primary anteroposterior configuration known as the “trap door” phenomenon, as well as secondary collapse due to hypopharyngeal latero-lateral occlusion or anterior-posterior collapse of the lymphatic lingual tonsil hypertrophy. Furthermore, the length of the glosso-epiglottic fold may determine the type of collapse noted, as would the bulk of the most posterior aspect of the tongue base. If there is a long glosso-epiglottic fold and therefore a lot more space between the base of the tongue and the epiglottis, then one would more likely encounter the trap-door phenomenon. Conversely, if the valecullae are extremely narrow with a short gloss-epiglottic fold and a shorter but tighter glosso-epiglottic fold, then the pattern of collapse would be different [28]. Additionally, an ary-epiglottis fold pattern of collapse may also be observed, which is similar to laryngomalacia in pediatric populations. Different approaches may be necessary to address each type of epiglottis collapse, and a careful selection of the therapy is mandatory based on the previously described causal mechanism (epiglottoplasty techniques, partial epiglottectomy). It is also important to note that arytenoid edema, often caused by esophageal-laryngeal reflux, may be a contributing factor in a laryngeal collapse during apnea events [29].

Finally, nasal pathology can induce mouth breathing and be of major importance in apnea/hypopnea pathophysiology [30,31]. When mouth breathing occurs, resistance in the UA increases and the posterior movement of the mandible occurs. It is speculated that on the one hand, nasal pressure receptors play a role in controlling the position of the soft palate, and on the other hand, the tension of the dilatative pharyngeal muscles decreases and triggers the UA collapse [32,33]. After nasal surgery (septoplasty, turbinoplasty, and FESS), changes in UA collapsibility were observed at different UA levels. Bosco et al. identified a significant change in UA collapse at the level of the hypopharynx in patients after nasal surgery. The authors also observed a significant increase in the number of patients without UA collapse as well as a decreased number of patients with partial and total collapse [34]. Consequently, nasal pathology should be reported in all DISE classification systems.

The VOTE classification does not make a distinction between pharyngeal collapse and obstruction, nor does it mention the grade of palatine tonsil hypertrophy, oral tongue collapse, or abnormalities in the nasal cavities. Moreover, the VOTE classification does not mention the response of the UA to specific maneuvers typically performed during DISE, such as jaw thrust, chin lift, oral appliance simulator application, head rotation, or body lateral positioning [7], highlighting the importance of specific notes in addition to VOTE scores in DISE final reports.

The importance of DISE in the therapeutic decision-making process for OSA patients has been reported in the literature, but with controversial results. Green et al. [35] conducted a multicenter DISE cohort study and reported that oropharyngeal latero-lateral collapse, without palatine tonsil hypertrophy, is associated with the poorest surgical outcomes. Similar results have been observed by Huyet et al. [23] in a multi-center DISE study on an OSA patient population selected for hypoglossal nerve stimulation. However, Certal et al. [36] reported a lack of evidence about the association between the impact of DISE on candidate selection for surgery and surgical outcomes in a systematic review of the comparison between awake examination vs. DISE for the surgical decision-making process. More recently, Lisan et al. [37] reported similar results in a systematic review and meta-analysis on the limited role of DISE in improving patient selection and surgical outcomes but remarked on the low level of evidence in the included studies, mainly due to the vast heterogeneity of pharyngeal soft tissue surgical procedures and the small patient population presented in each single study.

Interestingly, Albdah et al. [38] in a systematic review and meta-analysis observed that DISE plays a significant role in changing the initial treatment surgical options in about fifty percent of OSA patients, particularly for epiglottis involvement and soft palate surgical procedures. The change in surgical treatment was more significant when midazolam was applied during DISE compared to other sedation protocols. Finally, Iannella et al. [39] conducted a recent single-center randomized controlled trial comparing functional results in patients treated with barbed reposition pharyngoplasty (BRP) surgery with and without a preoperative DISE evaluation, achieving an 83% surgical success rate in the preoperative DISE group versus a 60% surgical success rate in OSA patients who underwent pharyngoplasty without preoperative DISE. These latest data highlight the importance of a unified DISE methodology and surgical procedure to analyze DISE results and the impact of surgical treatment outcomes.

Moreover, DISE could improve insight into CPAP failures by revealing a primary epiglottis collapse (trapdoor phenomenon), which represents one of the causes of CPAP failure. DISE can also improve the understanding of low CPAP compliance by observing nasal cavity abnormalities leading to mouth breathing, which should always be reported in DISE analysis [40].

Therefore, the methodology used to perform DISE and the interpretation of its findings are of pivotal importance. There are still significant differences in several studies on DISE application in surgical candidate selection, particularly regarding the modality for the detection of the level of sedation (Ramsay modified score of 5 vs. 60–80 BIS level of sedation) and the modality of sedative agents’ infusion (bolus vs. TCI technique) [41,42,43]. All these aspects should be considered both when the positive impact of DISE on surgical outcomes is reported and when the limited predictive value of DISE is discussed.

The final classification of level, grade, and patterns of UA collapse in DISE represents the most crucial step of the entire procedure because all treatment options may be determined based on the site of collapse reported, which does not necessarily represent the underlying causal mechanism.

While the VOTE classification has the main positive aspect of being widely adopted for its simplicity, it limits patient selection for appropriate surgical and non-surgical treatment options by segmenting the airway in sites of collapse, missing the whole understanding of how an increased UA collapsibility affects multiple sites simultaneously.

The main issue about the low predictive value of DISE for surgical outcomes is that surgical outcomes are not always associated with VOTE classified DISE findings. As reported above, Green et al. highlighted that in 35% of patients who underwent isolated palatal surgery after DISE [35]. A recent literature review on predictors of the success of pharyngeal surgery in OSA treatment concluded that there is a lack of an accurate protocol for the indication of pharyngeal surgery [44]. Our goal for the current review was to obtain comprehensive literature data on DISE. Based on the findings, we were able to emphasize that the final report and scoring classification system used in DISE is not universally accepted and does not cover all patterns of collapse and obstruction within the UA, which is critical for decision-making in sleep surgery. For example, an oblique palatal phenotype is favorable for performing reposition pharyngoplasty like Barbed Reposition Pharyngoplasty, whereas [45], a vertical palatal phenotype is not, and other procedures should be considered like transpalatal advancement [24]. Using VOTE classification, it is impossible to obtain this information. Therefore, we believe that the literature review we performed brings a new insight into our evaluation of upper airways in OSA patients.

It is crucial to have a detailed and descriptive DISE classification to ensure objective identification of the causal mechanism of increased UA collapsibility in order to improve patient selection for the many different PAP-alternative treatment options for OSA patients. This classification should take into account objective measures of collapsibility affecting all levels of the UA (multilevel collapse), including the nasal cavities, and compare the awake assessment of the UA during tidal breathing with the mouth open and closed. The Mueller maneuver, which has limited validity, should be replaced with more accurate methods of analyzing tidal breathing, flow, and pressure, as demonstrated in a promising study by the Vanderveken and Dedhia groups [21,46].

## 5. Conclusions

OSA treatment overall strategy is steadily moving from a CPAP-centered “one-size-fits-all” approach to tailored multimodality treatment. Patients with high UA collapsibility are the best candidates for CPAP, with an adjunctive or salvage role for surgical treatment and oral appliances. Otherwise, OSA patients with mild-to-moderate UA collapsibility may be a good candidate for UA surgical treatment. However, more than 70% of OSA patients also have concomitant altered anatomical factors and non-anatomical factors (LG, AT, and neuromuscular impairment), with different impacts in each patient. Therefore, these patients may benefit from anatomical or non-anatomical treatments, such as UA surgery, MAD, and hypoglossal nerve stimulation.

DISE enables the three-dimensional and dynamic visualization of the UA during sleep, which is useful in selecting the best treatment option for OSA patients, particularly for surgical procedures. The assessment of the UA in the awake and asleep states can significantly differ in all the main pharyngeal levels involved in hypopnea or apnea events. A considerable amount of literature focuses on comparing DISE with natural sleep, interobserver reliability, decision-making processes for treatment options, and its predictive impact on surgical outcomes. However, a unified DISE methodology is not yet a reality. A final report and scoring classification system is not universally accepted, and it does not cover all patterns of events that occur simultaneously during the endoscopic examination. Furthermore, the feasibility of video-recording systems during DISE and/or real-time online cardiorespiratory monitoring would improve the interobserver agreement and increase the accuracy of DISE by adding objective measures of UA collapsibility and the identification of nonobstructive events (i.e., central apneas). The observation of the dynamics (i.e., severity of obstruction and multilevel patterns of collapse) seems to be meaningful for the comprehension of the underlying causal mechanism of UA obstruction [46].

Finally, OSA patients could undergo DISE with airflow monitoring and nasal positive airway pressure titration. Consequently, it is possible to determine the visual and airflow-based levels of pharyngeal opening pressure (PhOP). The DISE-PhOP evaluation can be performed visually or on an integrated recording platform to acquire endoscopic images, flow, and effort (belts or catheters) from the patient’s UA. A detailed description of the DISE-PhOP setup has been previously published [46]. In short, the nasolaryngoscope was passed through a custom-fitted mask into the nasal cavity. PAP (S9 VPAP, ResMed Inc., San Diego, CA, USA) was applied and titrated using a nasal mask (Pulmodyne, Indianapolis, IN, USA). Propofol anesthesia was administered to achieve sedation with a target Bispectral Index (BIS) of 50–70. Once a stable sedation plan is achieved, PAP titration starts. PhOP is derived from DISE-PAP visually by the elimination of inspiratory effort or from pressure-flow relationships, as previously described [46]. PAP stars at 4 cm H_2_O after respiratory events were observed at atmospheric pressure, with further titration at increments of 1 cm H_2_O at the termination of obstructive apnea or hypopnea. The evidence so far is that patients with a PhOP of less than 8 have a better response to hypoglossal nerve stimulation therapy, suggesting a tongue-driven mechanism. Preliminary data suggests that higher values of PhOP respond better to expansion pharyngoplasty and Maxillo-mandibular advancement. Visual DISE-PhOP could represent a method to measure UA collapsibility and could be applied routinely with significant interrater reliability [21].

Hopefully, in the future, the analysis of objective data with a more homogenous DISE methodology will allow for a better understanding of the role of DISE in patient selection and the comparison of surgical outcomes.

## Figures and Tables

**Table 1:** Results and papers selection of the extensive bibliographic research.

Total Papers Found on PubMed	Systematic Reviews, Meta-Analysis, RCTs	Papers Included in the Manuscript
105	54	46

**Table 3:** Main DISE indication.

1. OSA patients are unable to accept or tolerate the pressure of CPAP titration [17]
2. OSA patients who failed previous UA surgery [18]
3. OSA patients considering non–CPAP treatment options for OSA, such as UA surgery, MAD, hypoglossal nerve stimulation, positional therapy, or combination therapy [9]
4. OSA patients with socially problematic primary snoring [7]
